# Acarbose Unveiled: A Breakthrough in Postprandial Hypotension Treatment

**DOI:** 10.7759/cureus.62378

**Published:** 2024-06-14

**Authors:** Steven Pham, Gregory Mock, Robert Camferdam

**Affiliations:** 1 Internal Medicine, White County Medical Center, Searcy, USA; 2 Nephrology, White County Medical Center, Searcy, USA

**Keywords:** blood pressure, elder, diabetes type 2, acarbose, postprandial hypotension

## Abstract

Postprandial hypotension (PPH) is characterized by a drop in blood pressure (BP) of at least 20 mmHg within 15 minutes to two hours after any meal. This phenomenon is observed in approximately half of patients with type 2 diabetes mellitus and can also affect otherwise healthy elderly patients. Prolonged instances of PPH can cause serious complications, including but not limited to dizziness, frequent falls, weakness, and even loss of consciousness. Nonpharmacologic interventions can help, such as discontinuing any exacerbating medications, increasing salt and water intake, adopting lifestyle modifications, and engaging in muscle tension-reducing exercises. When these strategies fail, pharmacological treatments may become necessary. Medications like midodrine (an alpha-adrenergic agonist) or droxidopa (a norepinephrine precursor) are commonly prescribed to help maintain BP. However, should BP persistently remain low despite these interventions, alternative therapies are explored. Acarbose, an antidiabetic medication, is an alpha-glucosidase inhibitor that targets pancreatic alpha-amylase and membrane-bound intestinal alpha-glucoside hydrolase. The inhibition slows glucose absorption, further reducing postprandial glucose blood concentrations. This case report presents the management of a 67-year-old woman with persistent PPH that is unresponsive to midodrine, atomoxetine, and sodium chloride tablets. The addition of acarbose to her regimen yields appropriate maintenance of BP after meals. The patient was able to be safely discharged home after.

## Introduction

The incidence of postprandial hypotension (PPH) is increasingly more common in the elderly population, especially those who are hospitalized or critically ill [1]. The severity of PPH is closely associated with a decrease in systolic blood pressure (BP) [2]. PPH can lead to serious complications, such as syncope, falls, strokes, or coronary events. Studies have even indicated that PPH can serve as a predictor for new cardiovascular events [3]. Various mechanisms have been proposed as the causes of PPH, with most pointing toward defects in autonomic function and the release of gastrointestinal hormones. Following meals, there is an increase in the visceral blood supply capacity, resulting in a reduction of blood volume for cardiac regurgitation [4]. In healthy individuals, the body compensates by activating the sympathetic nervous system, which increases cardiac output through elevated heart rate and stroke volume [4]. However, as people age, the sympathetic system may not be as robust, leading to a decrease in baroreflex sensitivity and weakening the compensatory effect of the cardiovascular system, ultimately resulting in PPH [5].

Elderly patients with diabetes are also more susceptible to PPH [6]. PPH in this patient population is attributed to the impaired autonomic nervous system resulting from inadequate blood glucose control [6]. Because PPH lacks a single proposed mechanism, managing the condition poses significant challenges, particularly in diverse patient populations [7]. Research has shown that α-glucosidase inhibitors, such as acarbose, play a role in mitigating postprandial BP fluctuations by inhibiting carbohydrate absorption in the small intestine, thereby slowing gastric emptying [8]. Acarbose, used in the treatment of type 2 diabetes mellitus, triggers the release of glucagon-like peptide-1 (GLP-1) [9]. GLP-1 delays intestinal absorption and reduces hyperglycemia after meals [10,11].

Beyond its primary application, acarbose holds the potential to indirectly regulate BP by influencing the composition of the gut microbiota. In a previous study conducted by Gentilcore et al., sucrose was utilized as a source of carbohydrates that was given through an intraduodenal infusion [11]. Following this, acarbose was administered, effectively reducing the fall in BP by slowing carbohydrate absorption and increasing heart rate through the stimulation of GLP-1. This action helps attenuate the rise in splanchnic blood flow, particularly in the superior mesenteric artery [12]. Acarbose emerges as a promising intervention in improving PPH by both delaying gastric emptying and inhibiting postprandial splanchnic perfusion, thus reducing the risk of age-associated diseases in the elderly [13]. Such findings present a compelling avenue for addressing the fluctuations in postprandial BP drop [8].

## Case presentation

A 67-year-old woman with a past medical history of PPH, type 2 diabetes mellitus, and multiple cancers (laryngeal cancer status post-radiation and breast cancer status post-chemotherapy and partial right mastectomy) presented for generalized weakness. On arrival, her initial vital signs were notable for a BP of 82/51 mmHg. A month prior to this admission, the patient was admitted to the critical care unit for septic shock secondary to a urinary tract infection. At that time, she was unable to tolerate oral intake. Any attempts to feed her failed because she became significantly hypotensive, with systolic BP dropping to the 80s mmHg and diastolic BP dropping to the 50s mmHg. She also could not tolerate tube feeding, so the decision was made for total parenteral nutrition (TPN). She was then discharged home with levofloxacin along with TPN. She was also started on midodrine (10 mg) three times a day at home to help improve her BP.

When she presented back this time, her BP was still low at 76/40 mmHg with a mean arterial pressure (MAP) of 52 mmHg, so she was fluid resuscitated. Her blood culture grew *Enterobacter cloacae*, so cefepime was started. Because of her persistent hypotension, she was started on norepinephrine and vasopressin. The source of her infection was presumed to be from the port that was used to administer TPN, so the general surgeon removed it. The blood culture after port removal showed no growth. We had difficulty weaning off the vasopressors at first because her MAP consistently stayed below 60 mmHg even with midodrine use. Therefore, hydrocortisone 50 mg every eight hours was started briefly for two days, and midodrine was increased to 10 mg four times a day. Afterward, her vasopressin was turned off appropriately, followed by the discontinuation of norepinephrine when her MAP was improved to 60 mmHg. The initial plan was to try to wean off her vasopressors and initiate TPN through her port because she only had one central access at that moment. However, TPN was no longer an option because her port was infected. Since she was able to tolerate oral intake, we decided to let her eat and monitor her BP. She was encouraged to eat and drink coffee in an attempt to improve her postprandial BP. The patient stated that at home, her BP dropped after meals but came back up after an hour. We then measured her BP on the day she attempted oral intake (we tentatively referred to this as “Day 1”). Her BP before breakfast was 106/72 mmHg, which dropped to 61/38 mmHg after the meal (Table 1). Similarly, her BP before and after lunch was 98/56 mmHg and 65/40 mmHg, respectively (Table 2). Her BP before and after dinner did not fluctuate much, consistently in the 95/56 mmHg and 89/61 mmHg ranges, respectively. However, there was also a drop of around 30 mmHg in BP after dinner over the next day (Table 3).

**Table 1:** BP measurement (in mmHg) before and after breakfast after the patient began oral intake Acarbose was added on Day 7. The patient was discharged on the morning of Day 9. BP, blood pressure

BP before breakfast (mmHg)	BP after breakfast (mmHg)	Difference in systolic BP (mmHg)	Difference in diastolic BP (mmHg)	Day from admission
106/72	61/38	45	34	Day 1
106/73	56/33	50	40	Day 2
119/90	70/43	49	47	Day 3
109/77	66/42	43	35	Day 4
93/66	69/42	24	24	Day 5
82/53	68/44	14	9	Day 6
105/68	101/60	4	8	Day 7
100/70	100/74	0	4	Day 8
85/57	81/53	4	4	Day 9

**Table 2:** BP measurement (in mmHg) before and after lunch after the patient began oral intake Acarbose was added on Day 7. BP, blood pressure

BP before lunch (mmHg)	BP after lunch (mmHg)	Difference in systolic BP (mmHg)	Difference in diastolic BP (mmHg)	Day from admission
98/56	65/40	33	16	Day 1
107/70	63/40	44	30	Day 2
93/66	61/39	32	27	Day 3
88/67	60/39	28	28	Day 4
Not documented, unknown reason	Not documented, unknown reason	Not applicable	Not applicable	Day 5
106/75	104/60	2	15	Day 6
80/50	70/50	10	0	Day 7
100/75	91/65	9	10	Day 8

**Table 3:** BP measurement (in mmHg) before and after dinner after the patient began oral intake Acarbose was added on Day 7. BP, blood pressure

BP before dinner (mmHg)	BP after dinner (mmHg)	Difference in systolic BP (mmHg)	Difference in diastolic BP (mmHg)	Day from admission
95/56	89/51	6	5	Day 1
103/69	67/41	36	28	Day 2
95/62	75/48	20	14	Day 3
114/83	93/59	21	24	Day 4
94/62	66/36	28	26	Day 5
92/59	90/56	2	3	Day 6
118/78	115/76	3	2	Day 7
96/71	86/66	10	5	Day 8

On Day 2, sodium chloride tablets of 1 gram twice a day were added, and fluid was started at a rate of 80 cc/hour. The patient was also given atomoxetine (10 mg) twice a day. BP measurements after this regimen were as follows, respectively shown as before and after each breakfast, lunch, and dinner: 109/73 mmHg and 56/33 mmHg, 107/70 mmHg and 63/40 mmHg, and 103/69 mmHg and 67/41 mmHg (Tables 1-3). When the maintenance fluid was discontinued, the patient was encouraged to drink at least 500 milliliters to 1 liter of water a day. The dose of atomoxetine was increased to 18 mg, and the frequency remained the same. On Day 7, we initiated a trial of acarbose with the instruction to take 20-30 minutes prior to meals. We started at a lower dose of 25 mg with each meal and titrated up to 100 mg. Since acarbose was initiated, we saw that the BP dropped about 10 mmHg in between meals. After the dose was increased, the drop in BP before and after each meal became almost nonexistent. The patient tolerated the medication well with no significant side effects. Her systolic BP was maintained in the 100s mmHg, with some readings even showing normal BP (Tables 1-3). Her MAPs were also improved to 60-70s mmHg with no significant drop. The patient was stable enough to be discharged home after.

## Discussion

Hypotension, commonly known as low BP, is a medical condition characterized by a decrease in the force exerted by the blood against the arterial walls [14]. While some cases of hypotension may be asymptomatic and benign, severe instances can result in dizziness, fainting, and even life-threatening complications. PPH is also more prevalent in patients with diabetes because of similar pathologies, such as autonomic dysfunction and delayed gastric emptying [6]. Nonpharmaceutical management strategies include eating more frequent and smaller meals, drinking water before meals, and exercising post-meal [15]. However, there is no single pharmaceutical treatment for PPH.

Managing PPH is particularly difficult for patients with type 2 diabetes mellitus. Any strategies that result in the slowing of gastric emptying and the absorption of small intestinal carbohydrates could be effective. One medication that has shown its therapeutic value in both diabetic and nondiabetic patients is acarbose. In individuals with PPH, acarbose has demonstrated a notable improvement in systolic BP, with an average reduction of approximately 15-20 mmHg post-intake. Acarbose functions by inhibiting alpha-glucosidase enzymes in the small intestine, slowing the breakdown of complex carbohydrates into simple sugars like glucose [16]. This action reduces the postprandial surge in blood glucose levels [6]. While acarbose is predominantly used to manage diabetes and enhance glycemic control, researchers have proposed its potential benefits in addressing hypotension [17]. The potential link between acarbose and hypotension is based on its ability to modulate the gut microbiota. Recent research indicates that the gut microbiota plays a crucial role in regulating BP through the production of various bioactive compounds [11,18]. By altering the gut microbiota composition, acarbose mitigates postprandial BP fluctuations by impeding the absorption of carbohydrates and slowing down the rate of gastric emptying.

Our patient initially received midodrine, atomoxetine, and sodium chloride tablets. These measures yielded marginal improvement in the postprandial BP drops. However, since the initiation of acarbose, the fall in BP after meals has improved significantly, decreasing by 10 mmHg or becoming nearly negligible. Despite the results seen in our patient, there is still a lack of clinical trials on the full potential effect of acarbose. While acarbose shows promise as a potential treatment for PPH, further clinical exploration is necessary to establish its efficacy, safety, and optimal dosing specifically for the treatment of PPH.

## Conclusions

PPH poses a common risk among older individuals, demanding careful consideration of its mortality and complications. Current treatments for this condition are notably suboptimal, presenting a challenge in finding effective solutions. This case report emphasizes the potential of alpha-glucosidase inhibitors like acarbose as a primary treatment for PPH, both in diabetic and nondiabetic patients. However, it is essential to acknowledge certain limitations, including the timing and methodology of BP measurements, which may introduce variables into the results. Further studies are crucial to gain a deeper understanding of acarbose’s effectiveness, especially in nondiabetic patients. Previous studies have suggested that acarbose may have the potential to reduce the postprandial fall in BP, a notion supported by our case. The positive outcomes observed in our study endorse the use of acarbose to reduce the duration and severity of PPH. It is crucial to conduct further studies to better comprehend the effectiveness of acarbose as a viable treatment option for PPH, especially across different patient populations, and to investigate if these effects are sustained during chronic administration.
